# Impute the missing data by combining retrieved dropouts and return to baseline method

**DOI:** 10.1371/journal.pone.0323496

**Published:** 2025-05-29

**Authors:** Xiaozhou Li, Zhenyu Yang, Chuanji Yuan, Jiaqing Liu, Zuojing Li

**Affiliations:** 1 Department of Pharmaceutical informatics, School of Shenyang Pharmaceutical University, Shenyang, Liaoning, China; 2 Department of Biostatistics and Data Analysis, Liaoning Yidu Medical Data Technology Co., Ltd., Shenyang, Liaoning, China; Max Planck Institute for Solid State Research, GERMANY

## Abstract

Currently, various methods have been proposed to handle missing data in clinical trials. Some methods assume that the missing data are missing at random (MAR), which means that it is assumed that subjects who stopped treatment would still maintain the treatment effect. In many cases, however, researchers often assume that the missing data are missing not at random (MNAR) to conduct additional sensitivity analyses. Under the MNAR assumption, whether using some conservative imputation methods such as RTB (return to baseline) method, J2R (jump to reference) method, and CR (copy reference) method, or optimistic imputation methods like multiple imputation (MI) and its derivative RD (retrieved dropout) method, biases compared to the true treatment effect can occur in some scenarios. This paper aims to propose a method that can impute results while considering the occurrence of intercurrent events, thereby reducing the bias compared to the true treatment effect. This method combines the RD method with the RTB formula, reducing the biases and standard errors associated with using either method alone. Considering the differing treatment effects between RD subjects and non-RD subjects, our imputation results often align more closely with the true drug efficacy.

## 1 Introduction

### 1.1 Current state of research

In randomized trials, missing data refers to the lack of subjects’ observations at scheduled time points, which is common in long-term studies with multiple follow-ups [1]. There are various reasons for missing data, including treatment termination, taking rescue medication, and missing visits [2]. The ICH E9(R1) guideline refers to unplanned events affecting the observational results in trials as ‘intercurrent events’ and proposes five strategies: treatment policy strategy, hypothetical strategy, composite variable strategy, while on treatment strategy, and principal stratum strategy. These strategies are different in how to handle data following intercurrent events. Some common statistical analysis methods, such as mixed-effects models for repeated measures (MMRM) [3], are often used under the assumption of missing at random (MAR), in which missing data depend only on observed data [4]. However, in clinical trials, this assumption may not always hold because subjects’ conditions after the trial discontinuation are often differ from those before the trial discontinuation. In addition, there is the MCAR (missing completely at random) missing data mechanism. MCAR refers to the situation where the data are missing completely at random, and the reason for the missing data is unrelated to all the data. However, this missing data mechanism is less commonly applied in clinical trials. Therefore, it is crucial to understand different handling strategies for missing data and select appropriate statistical analysis methods for ensuring the accuracy and reliability of randomized trial results.

Considering various types of missing data, researchers need to assume data are missing not at random (MNAR) and conduct additional sensitivity analyses. Under the MNAR assumption, missing data are not only dependent on observed data but also related to the unobserved missing values [5]. Multiple imputation (MI) methods are designed to handle MNAR-type missing data and are considered a preferred approach for dealing with missing data due to their applicability to both MAR and MNAR types [6,7]. Unlike mixed-effects models for repeated measures (MMRM), which relies on a specific model to handle missing data, MI involves imputing missing values based on the distribution and imputation model defined by the user and utilizing the characteristics of the complete data set to estimate the missing values [8]. However, when subjects early determinate treatments due to intercurrent events (such as adverse reactions or lack of efficacy), imputed result may only reflect efficacy of situation without intercurrent events. Those results potentially lead to overestimate the treatment efficacy and bias of the true efficacy [9].

In order to estimate treatment efficacy more precisely, conservative missing data handling methods are used, such as J2R (jump to reference) and CR (copy reference) methods. These methods assume that subjects are no longer benefit from the trial treatment after treatment terminations. Moreover, the expected results of the treatment group after termination would be similar to those of the control group after adjusting for baseline characteristics [10]. Considering the treatment impact, the RTB (return to baseline) method is a more conservative imputation approach. It assumes that the treatment efficacy will return to baseline levels after the treatment termination (i.e., non-improvement) [11]. This assumption sometimes aligns with real-world scenarios. Unlike the traditional baseline observation carried forward (BOCF) method, the RTB method assumes that values of post intercurrent events have the same distribution as the baseline but are not necessarily equal to each subjects’ baseline value, which introducing variability and uncertainty into the imputed results [12,13]. However, the RTB method is more suited for situations that subjects are on placebo or the half-life of drug exposure is short.

The retrieved dropout (RD) method has been proposed to balance between the RTB method and multiple imputation (MI) method [14]. It adheres to the intention-to-treat (ITT) principle in clinical trials by using data from the subjects who terminate the treatment but continue to provide their data, to impute missing data for other subjects. Thereby it offers a more accurate estimation of drug efficacy [15,16]. In this study, we refer to subjects who withdrawn treatment but continue to provide data as RD subjects, and those who withdrawn treatment and no longer provide data as non-RD subjects. For non-RD subjects, data are considered missing after subjects withdraw treatment. The RD method is primarily applied in treatment policy strategy scenarios, if the study aims to estimate the treatment policy estimand, the RD method can be used [17]. Under this strategy, the RD method has been shown to have advantages in reducing bias compared to other imputation methods [18]. However, the RD method also has limitations, especially when the types of intercurrent events in RD subjects differ from non-RD subjects, which may impact the accuracy of imputation and lead to biased efficacy estimates. Therefore, if the types of intercurrent events are clear or efficacy data before the treatment termination can be collected, researchers need evaluate the most suitable imputation method to achieve a reasonable balance between conservative and optimistic efficacy estimates. Based on the limitations and characteristics of these methods, we proposed a new improved method for handling missing data, aiming to estimate treatment efficacy in clinical trials more accurately. In this study, we only consider the scenario where non-RD subjects do not return to the trial after withdrawal treatment, meaning that all data for non-RD subjects after withdrawal are considered missing, non-RD subjects withdraw from both treatment and the trial at the same time. In addition, we only considered the scenario where all data for RD subjects could be collected after they withdraw treatment, meaning that all data for RD subjects after withdrawal are considered not missing, RD subjects withdraw treatment, they did not withdraw from the trial.

## 1.2 Method

### 1.2.1 Return to baseline (RTB) method.

The RTB method assumes that the treatment efficacy is eliminated after the treatment termination. The conditional predictive distribution of missing data depends only on the baseline value. The conditional predictive distribution for these subjects is given by:


$$\begin{array}{c} P\left(Y_{J}^{mis}|\theta^{*},Y_{0}^{obs},X\right) \end{array}$$ (1)

where *j* is the visit index, where *j* = 0 represents the baseline visit and *j* = *J* represents the final visit in the study. j=0,⋯,J*.* $Y_{J}^{mis}$ is the imputed value of the missing data at the *J*^th^ visit, $Y_{0}^{obs}$ is the baseline value, $\theta^{*}\;$is a randomly drawn unknown parameter from the posterior distribution of the observed data, and X is covariate matrix consisting of variables that are correlated with $Y_{J}$ or plausibly affect the missingness of $Y_{J}$.

The RTB method is different from the traditional regression-based baseline observation carried forward (BOCF) method. The RTB method imputes values that have the same distribution as the baseline values but are not necessarily equal to each individual subjects’ baseline value. This introduces internal variability and flexibility, as well as uncertainty due to random error [19].

However, Qu and Dai demonstrated that the traditional RTB method may be biased or exhibit variance inflation when the probability of missing data depends on observed baseline or intermediate results after baseline. To address this issue, a new RTB formula has been proposed.


$$\begin{array}{c} Y_{iJ}^{\left(imp*\right)}=Y_{iJ}^{\left(imp\right)}-\overset{―}{Y_{iJ}}+\overset{―}{Y_{0}} \end{array}$$ (2)

Where *i* is the subject index. $Y_{iJ}^{\left(imp\right)}$ is the imputed value of *i*^*t*h^ subject with missing data at the *J*^th^ visit, i=1,⋯I; j=0,⋯,J; is the mean of the imputed values at the last visit, and $\overset{―}{Y_{0}}$ is the mean of the baseline values.

When the expected change in missing data is relative to the baseline is close to zero, the RTB method can be a feasible and simpler option for imputation.

### 1.2.2 The retrieved dropout (RD) method.

The RD method assumes that RD subjects and non-RD subjects in the same treatment group have the same efficacy. The conditional predictive distribution for non-RD subjects is given by:


$$\begin{array}{c} P\left(Y_{J}^{mis}|\theta^{*},Y_{RD}^{obs},X\right) \end{array}$$ (3)

Where *j* is the visit index, where *j* = 0 represents the baseline visit and *j* = *J* represents the final visit in the study. j=0,⋯,J*.* $Y_{J}^{mis}$ is the imputed value of the missing data at the *J*^th^ visit, $Y_{RD}^{obs}$ is the observed data of the RD subjects, $\theta^{*}\;$is a randomly drawn unknown parameter from the posterior distribution of the observed data, and X is covariate matrix consisting of variables that are correlated with $Y_{J}$ or plausibly affect the missingness of $Y_{J}$.

Primarily based on the principle of multiple imputation, the RD method uses RD subjects’ to establish an imputation model and fill in the missing data. It is more suitable for treatment policy strategy. But it cannot be applied to all imputation scenarios of treatment policy strategy. For instance, if RD subjects terminate treatment due to adverse events, whereas non-RD subjects drop out due to lack of efficacy, adverse events do not equate to lack of efficacy. In such cases, treating non-RD subjects as if they experienced adverse events often leads to an wrong estimation of the true efficacy. Therefore, the RD method leads to bias because of involving different intercurrent events.

### 1.2.3 RD-RTB.

Due to limitations of the RTB method and RD method, we integrated these two methods and designed an new method to account for different types of intercurrent events, which can provide lower bias under the treatment policy strategy.

## 2 The principle of RD-RTB method

First, let $Y_{ij}$ be the assessment value of the *i*^th^ subject with missing data at the *j*^th^ visit. The study endpoint of the clinical trials is the changes from baseline (i.e., visit 0) at the final visit (i.e., visit J) with the formula as follows:


$$\begin{array}{c} \Delta_{i}=Y_{iJ}-Y_{i0}\# \end{array}$$ (4)

Where *i* is the subject index, *j* is the visit index, where *j* = 0 represents the baseline visit and *j* = *J* represents the final visit in the study. i=1,⋯I;j=0,⋯,J*.*

Normally, baseline values are not missing (i.e., $Y_{i0}$ is not missing), while some subjects may have missing at visit J (i.e., $Y_{iJ}$ is missing for some i). By using the RTB method, we assume that subjects have no retained efficacy, making the handling of visit endpoints particularly conservative [20]. However, the extent of retained efficacy also does not necessarily depend entirely on the efficacy of RD subjects. Therefore, using the RD method for imputation often does not closely approximate the true efficacy, leading to bias from the true value.

For this situation, we modify the RTB method formula. The RTB formula is as follows:


$$\begin{array}{c} Y_{iJ}^{\left(imp*\right)}=Y_{iJ}^{\left(imp\right)}-\overset{―}{Y_{iJ}}+\overset{―}{Y_{0}} \end{array}$$ (5)

$$\begin{array}{c} \begin{array}{c} \beta_{i}=\overset{―}{Y_{0}}-\overset{―}{Y_{iJ}} \end{array} \end{array}$$ (6)

Where $\beta_{i}$ is a correction term for the *i*^th^ subject after using RTB method. We make modifications to the correction term $\beta_{i}$, The formula is as follows:


$$\begin{array}{c} \begin{array}{c} \alpha_{i,0}=\beta_{i}-\left(1-e^{-\frac{\sqrt{Y_{i0}-\min_{i}Y_{i0}}}{\frac{T}{t_{i}}}}\right)\times \beta_{i} \end{array} \end{array}$$ (7)

Where $\alpha_{i,0}$ is is the result of applying weights to $\beta_{i,0}$, in the formula, the exponential function is a value between 0 and 1, and may approach 0 infinitely closely. ${\;t}_{i}$ is the actual duration of exposure Time for the *i*^th^ subject, T is the expect duration of exposure time of the clinical trial, $Y_{i0}$ is the baseline value of different subject with missing data, $Y_{i0(min)}$ is the minimum baseline value among all non-RD subjects.

This method can estimate values near the RD imputation results and adjust for overestimation or underestimation based on different types of concurrent events, making the imputation results closer to the efficacy results of the true dataset.

The formula includes several additional variables compared to the RTB formula. After applying the exponential function transformation, we obtain a value between 0 and the RTB method correction term. We can see that $\alpha_{i,0}$ is a part of the original correction term of the *i*^th^ subject. To address different intercurrent events, we further modify $\alpha_{i,0}$ by adjusting its sign, allowing the final imputation result to be either higher or lower than the RD method imputation result to accommodate various intercurrent events. The determination of the sign of $\alpha_{i,0}$ is based on the efficacy rate of the subject before exiting the treatment. The efficacy rate formula is as follows:


$$\begin{array}{c} \begin{array}{c} V_{rd}=\frac{\sum_{k=1}^{K}\Delta_{k}}{\sum_{k=1}^{K}V_{k}} \end{array} \end{array}$$ (8)

$$\begin{array}{c} \begin{array}{c} V_{mis}=\;\frac{\sum_{i=1}^{I}\Delta_{i}}{\sum_{i=1}^{I}V_{i}} \end{array} \end{array}$$ (9)

Where k is different RD subject, k=1,2,3,…,K;i is different non-RD subject, $i=1,2,3,\ldots ,I;\Delta_{k}$ is the change of *k*^th^ RD subject from baseline to the visit immediately before the subject exits treatment (for remedial medication subjects, it is the maximum change from baseline among all subjects who exited treatment). $\Delta_{i}$ is the change of *i*^th^ non-RD subject from baseline to the visit immediately before the subject exits treatment (for remedial medication subjects, it is the maximum change from baseline among all subjects who exited treatment). $V_{k}$ is the duration of *k*^th^ RD subject participated in visits from baseline to just before exiting treatment. $V_{i}$ is the duration of *i*^th^ non-RD subject participated in visits from baseline to just before exiting treatment.


$$\begin{array}{c} \rho =V_{rd}-V_{mis} \end{array}$$ (10)

$$\begin{array}{c} sign(\rho )=\pm 1 \end{array}$$ (11)

Where ρ is the relative efficacy of non-RD subjects compared to RD subjects just before exiting treatment. $V_{mis}$ is the overall efficacy rate of the change from baseline to the visit immediately before exit for all non-RD subjects, $V_{rd}$ is the overall efficacy rate of the change from baseline to the visit immediately before exit for all RD subjects.

These values reflect the severity of the impact of intercurrent events on subjects. The sign of the ρ value determines the value of sign(ρ), They represent situations where non-RD subjects have better or worse efficacy compared to RD subjects. This approach allows for adjustment of the sign of the correction term to address different occurrences of intercurrent events. It enables flexible adjustments to the final estimate, resulting in either an optimistic or conservative estimate, thereby bringing it closer to the true efficacy.

In clinical trials evaluating drug efficacy, the drug’s effectiveness is often assessed by comparing the differences between the treatment group and the control group. When analyzing these group differences, results from the RD method and the RTB method can differ significantly.

Using the RD method, since there is a certain correlation between the efficacy of RD subjects and the drug efficacy, the imputed results for non-RD subjects are also related to the drug efficacy. This correlation means that the bias in RD method’s group differences does not fluctuate significantly with changes in drug efficacy, making the RD method’s estimates more stable relative to the true efficacy.

In contrast, the RTB method regresses imputed values back to baseline values, making the imputed values completely independent of the drug efficacy. As the drug efficacy increases, the bias of the RTB method from the true efficacy tends to grow, leading to greater fluctuations in the group differences compared to the true efficacy. This makes the RTB method less reliable for drawing accurate conclusions.

Based on the characteristics of the RTB formula, we have made further modifications to the $\alpha_{i,0}$, The formula is as follows:


$$\begin{array}{c} \begin{array}{c} \alpha_{i}=sign(\rho )\times \left|1-\frac{\varphi_{0}}{\varphi_{1}}\right|\times \alpha_{i,0} \end{array} \end{array}$$ (12)

Where $\alpha_{i}\;$ is the ith subject’s result of applying weights to the correction term $\alpha_{i,0}$$,\varphi_{0}$ is the mean of absolute change from baseline to the last visit for subjects who withdrew from treatment after imputation using the RD method, $\varphi_{1}$ is the mean of absolute change from baseline to the last visit for subjects who did not experience intercurrent events.

To avoid significant bias in group differences as efficacy changes, we apply weights to the $\alpha_{i}$ values to prevent large fluctuations in the correction term with changes in efficacy. we use γ and δ for the treatment or placebo groups, respectively, where $\alpha_{1}$ and $\alpha_{2}\;$ are related as follows:


|γ|>|δ|

Where γ is the larger mean absolute value of $\alpha_{i}$, and different data processing is performed for the subjects in the two groups, according to the formula below:


$$\begin{array}{c} \begin{array}{c} \delta_{group}:\;Y_{iJ}^{\left(imp*\right)}=Y_{iJ}^{\left(imp\right)}+\alpha_{i} \end{array} \end{array}$$ (13)

$$\begin{array}{c} \begin{array}{c} \gamma_{group}:\;Y_{iJ}^{\left(imp*\right)}=Y_{iJ}^{\left(imp\right)}+\alpha_{i}+\left(1-e^{-\frac{\sqrt{Y_{i0}-\min_{i}Y_{i0}}}{\frac{T}{t_{i}}}}\right)\times \left(\gamma -\alpha_{i}\right) \end{array} \end{array}$$ (14)

Where $\gamma_{group}$ is the group where the mean of $\alpha_{i}$ is γ, $\delta_{group}$ is the group where the mean of $\alpha_{i}$ is δ.

Using the above formula, we ensure that the group differences remain more stable and closer to the RD method’s differences as efficacy fluctuates. Our formula correlates the imputed results with the subjects’ baseline values. When performing ANCOVA (analysis of covariance) on each imputed result using least squares means to estimate efficacy,this approach enhances the correlation between the imputed results and baseline values, leading to smaller standard errors. Ultimately, integrating results using rubin’s rules also reduces the standard error of the group efficacy differences. Furthermore,when comparing group differences,the results exhibit flexible adjustments based on RD method results, making them closer to the true efficacy. Additionally, as drug efficacy changes, the stability of bias influenced by drug efficacy is more stable compared to the RTB method.

## 3. Simulation study

### 3.1 Simulation procedure

In order to verify the rationality and effectiveness of our improved method, we conducted a simulation of a clinical trial. This is an investigation into the efficacy of drug treatment for subjects suffering from depression, using HAMD-17 (hamilton depression rating scale) as the endpoint to assess the efficacy of subjects. HAMD-17 is a widely validated and applied depression assessment tool that comprehensively evaluates the depressive symptoms of patients, including emotional, physical, and cognitive manifestations, The 17 items of the HAMD-17 assess different symptoms of depression, with each item typically scored on a scale of 0–4 or 0–2. The items include, but are not limited to, depressed mood, suicidal ideation, difficulty falling asleep, etc. The total score ranges from 0 to 52. [21]. In this clinical trial simulation, we divided the subjects into a treatment group and a placebo group, simulated the depressive rating data of the subjects, and compared the changes before and after treatment between the two groups. By comparing the efficacy of the two groups, we can objectively evaluate drug efficacy to verify the rationality and effectiveness of our improved method.To implement our clinical trial simulation process, we used R package-simcausal to realize the data collected in this clinical trial. The R package-simcausal is a tool specially designed for simulating longitudinal data in clinical trials, which provides rich features and flexibility, especially emphasizing the types of data and interventions often encountered in real-world causal inference problems. Through simulating the distribution of data (normal distribution, bernoulli distribution, etc.), generating datasets that conform to the data structure of clinical trials with multiple visits can help us simulate complex research designs and data structures [22]. Our process for simulating data is as follows:

1. An 8-week clinical trial with biweekly collections of HAMD-17 ratings from subjects will be simulated by using the R package, simcausal. We have selected subjects with HAMD-17 scores ranging from 15 to 35 for inclusion in this study, based on the actual depressive conditions of most clinical trial subjects and in conjunction with the study by Asghar et al. [23]. The baseline HAMD-17 scores was simulated from a normal distribution with a mean of 25 and a standard error of 5. The subjects were randomly divided into a treatment group and a placebo group. In the treatment group, subjects experienced an average decrease of 2.5 in HAMD-17 scores per visit, while in the placebo group, the average decrease was 0.5 per visit. Additionally, each visit’s decrease results followed a normal distribution with a standard error of 0.5. Based on such a data distribution, we simulated data for 800 subjects (400 subjects in treatment group and 400 subjects in placebo group).
2. In real clinical trials, subjects often withdraw from the treatment due to various intercurrent events, such as adverse events, disease progression, or personal reasons. To simulate a real-world clinical trial, the R package, MICE was used to set 30% of the subjects in the generated complete dataset as withdrawn from treatment, including both RD subjects and non-RD subjects. We set the proportions for subjects who withdraw treatment at different visits, the proportions of subjects who withdrawn treatment at the second, third, and fourth visits were set at 1:1:1.The subsequent visits for withdrawn treatment subjects were set as missing.
3. Due to the Treatment policy strategy used in this trial, we will make every effort to collect data from each subject who withdraws from treatment. However, considering factors such as costs and the personal wishes of the subjects, it is still inevitable that some data from subjects who have withdrawn from treatment will remain missing. Considering the relatively low proportion of RD subjects,we set 20% of the withdrawn treatment subjects as RD subjects, meaning they continue to have their data collected after withdrawal treatment. For their missing visit, we assigned new observed values. We assigned different weights to the efficacy of each subject before withdrawal treatment (the change from baseline at the last visit before withdrawal treatment) to represent the different levels of efficacy retained by the subjects after withdrawal treatment.Different weights were set to generate new complete datasets. For RD subjects in the treatment group, we assume that these subjects retain a portion of their efficacy after withdrawal treatment, with weight values set at 10%, 30%, 50%, 70%, and 90% of the efficacy before withdrawal treatment. For RD subjects in the placebo group, we assume that they will adopt remedial treatment measures after withdrawal treatment, with values set at 150% of the efficacy before withdrawal treatment.
4. To achieve difference in the efficacy between non-RD subjects and RD subjects, we retain all RD subjects among the withdrawn treatment subjects and analyze the pre-withdrawal efficacy rate (referring to equation 8 and 9) of each withdrawn subject, sorting them accordingly. We then select the top 70% efficacy rate subjects from the treatment group and the lowest 70% efficacy rate subjects, comparing the rate difference between the two groups as the subsequent simulated efficacy rate increase or decrease. First, we simulate the scenario where $\varphi_{0}<\varphi_{1}$. We use the R package- simcausal to simulate data for non-RD subjects, with the same data distribution as before, improving or reducing the efficacy rates of non-RD subjects to differentiate them from RD subjects. Again, we use the MICE package for missing data treatment for non-RD subjects, with a 100% missing rate and a 1:1:1 proportion of non-RD subjects for the second, third, and fourth withdrawal visits. We replace the data generated by the MICE package for non-RD subjects with this portion of the data, generating the final missing data set. For generating the real data set with complete data, We adopted the following approach. The efficacy settings for non-RD subjects are similar to those for RD subjects, we reassign values for the visits after withdrawal treatment, setting the assignment proportion at 100%. The treatment group retains 10%, 30%, 50%, 70%, and 90% of the pre-withdrawal efficacy, while the placebo group’s rescue treatment efficacy remains at 150% of the pre-withdrawal efficacy. We combine the data sets corresponding to withdrawn subjects retaining different efficacy levels with the data set of subjects without intercurrent events to form the complete data set which it can provide the real efficacy result. Similarly, we simulate the scenario where $\varphi_{0}>\varphi_{1}$. As in the case where $\varphi_{0}<\varphi_{1}$, we retain 110%, 120%, 130%, 140%, and 150% of the pre-withdrawal efficacy for the treatment group, while the placebo group’s rescue drug efficacy remains at 150% of the pre-withdrawal efficacy, to ensure that the efficacy of withdrawn subjects is higher than that of subjects without intercurrent events.

### 3.2 Simulation scenarios

To account for varying efficacy among different types of subjects, we have set up several scenarios.

 Scenario 1: The efficacy of subjects who withdrew from treatment is lower than that of subjects who did not experience intercurrent events and the efficacy of the non-RD subjects is higher than that of the RD subjects

 Scenario 2: The efficacy of subjects who withdrew from treatment is lower than that of subjects who did not experience intercurrent events and the efficacy of the non-RD subjects is lower than that of the RD subjects

 Scenario 3: The efficacy of subjects who withdrew from treatment is higher than that of subjects who did not experience intercurrent events and the efficacy of the non-RD subjects is higher than that of RD subjects

 Scenario 4: The efficacy of subjects who withdrew from treatment is higher than that of subjects who did not experience intercurrent events and the efficacy of the non-RD subjects is lower than that of RD subjects

The different scenarios set up in the simulation trial represent various situations in which subjects withdraw from treatment. In scenarios one and two, subjects who withdraw from treatment may do so due to lack of efficacy, occurrence of adverse events, and unwillingness to receive treatment, resulting in efficacy that is inferior to that of subjects who do not experience intercurrent events. In scenarios three and four, subjects who withdraw from treatment may do so because they take other medications after withdrawal or because of good efficacy, resulting in efficacy that is superior to that of subjects who do not experience intercurrent events. The difference in efficacy between RD subjects and non-RD subjects may be caused by different types of intercurrent events or different efficacy before withdrawal from treatment, among other reasons.

## 4. Results

**Fig 1:**
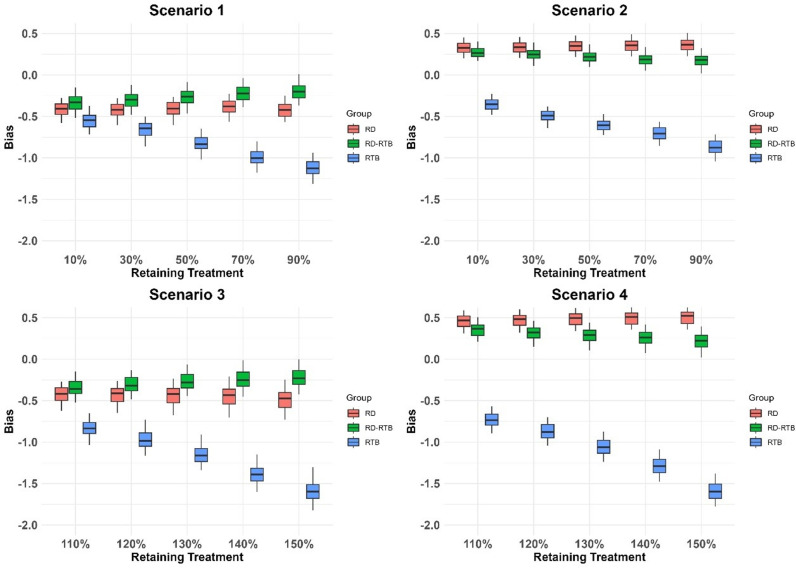
The distribution of bias for several imputation methods across 100 datasets and under different efficacy conditions.

**Fig 2:**
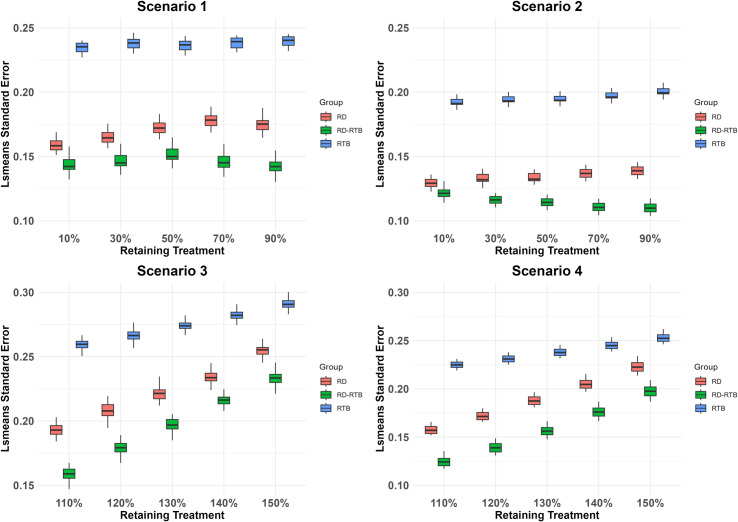
The distribution of lsmeans standard error for several imputation methods across 100 datasets and under different efficacy conditions.

**Fig 3:**
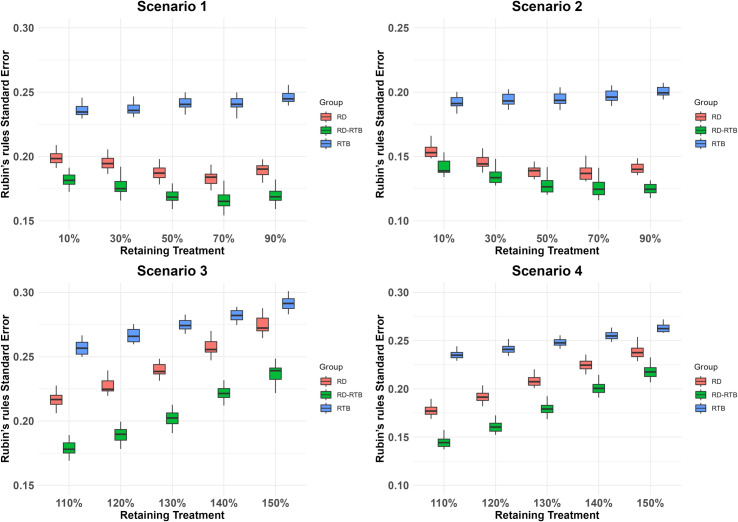
The distribution of rubin’s rule standard error for several imputation methods across 100 datasets and under different efficacy conditions.

In the simulation study, 20% of the withdrawn subjects are randomly selected as RD subjects. We generate 100 datasets following the steps outlined for the simulated dataset. For each final merged dataset, we use ANCOVA to estimate the treatment effects under different scenarios for each imputation result and apply Rubin’s rules to combine the results from 100 imputations for each dataset. We demonstrate the advantages of the improved algorithm using the bias between the efficacy estimates and the true efficacy, the standard error of the least squares means, and the standard error combined using Rubin’s rules. Positive (or negative) bias indicates that the imputation method may overestimate (or underestimate) the true treatment effect. To present our analysis results, we used boxplots to summarize the 100 datasets’ results of bias (Fig 1), least squares means standard error (Fig 2), and the standard error combined using Rubin’s rules (Fig 3) after performing ANCOVA analysis on 100 datasets in each scenario.We also calculated the average of the results from the analysis of the 100 datasets in each scenario (Tables 1–3).

**Table 1:** Bias - Comprehensive analysis results of several methods.

Scenario	Retained Efficacy				
Methods	10%	30%	50%	70%	90%
**Scenario 1**					
RD	-0.4044	-0.4053	-0.4098	-0.3915	-0.3999
RD-RTB	-0.3004	-0.2728	-0.2497	-0.2170	-0.1862
RTB	-0.5607	-0.6956	-0.8291	-0.9619	-1.0949
**Scenario 2**					
RD	0.3219	0.3367	0.3465	0.3521	0.3662
RD-RTB	0.2717	0.2562	0.2365	0.2172	0.1982
RTB	-0.1909	-0.3600	-0.5301	-0.7000	-0.8700
**Scenario 3**					
RD	-0.3649	-0.3628	-0.3685	-0.3737	-0.3843
RD-RTB	-0.3028	-0.2830	-0.2671	-0.2293	-0.2183
RTB	-0.8308	-0.9732	-1.1570	-1.3890	-1.5940
**Scenario 4**					
RD	0.4597	0.4720	0.4860	0.4981	0.5085
RD-RTB	0.3688	0.3361	0.3068	0.2786	0.2395
RTB	-0.7299	-0.8703	-1.1577	-1.3845	-1.5906

**Table 2:** LSM Std Err-Comprehensive analysis results of several methods.

Scenario	Retained Efficacy				
Methods	10%	30%	50%	70%	90%
**Scenario 1**					
RD	0.1653	0.1709	0.1755	0.1799	0.1769
RD-RTB	0.1489	0.1464	0.1461	0.1477	0.1484
RTB	0.2360	0.2365	0.2379	0.2404	0.2438
**Scenario 2**					
RD	0.1328	0.1359	0.1385	0.1405	0.1420
RD-RTB	0.1202	0.1180	0.1153	0.1146	0.1139
RTB	0.1926	0.1933	0.1949	0.1973	0.2006
**Scenario 3**					
RD	0.1893	0.2087	0.2207	0.2376	0.2576
RD-RTB	0.1627	0.1789	0.1957	0.2160	0.2310
RTB	0.2596	0.2665	0.2741	0.2824	0.2913
**Scenario 4**					
RD	0.1574	0.1719	0.1879	0.2051	0.2232
RD-RTB	0.1243	0.1388	0.1562	0.1759	0.1975
RTB	0.2150	0.2211	0.2277	0.2349	0.2466

**Table 3:** Rubin Std Err - Comprehensive analysis results of several methods.

Scenario	Retained Efficacy				
Methods	10%	30%	50%	70%	90%
**Scenario 1**					
RD	0.2019	0.1931	0.1873	0.1850	0.1903
RD-RTB	0.1851	0.1808	0.1768	0.1765	0.1808
RTB	0.2371	0.2377	0.2393	0.2418	0.2453
**Scenario 2**					
RD	0.1543	0.1479	0.1419	0.1419	0.1453
RD-RTB	0.1489	0.1395	0.1325	0.1286	0.1288
RTB	0.1934	0.1941	0.1958	0.1983	0.2016
**Scenario 3**					
RD	0.2155	0.2336	0.2434	0.2583	0.2762
RD-RTB	0.1802	0.1913	0.2032	0.2239	0.2458
RTB	0.2615	0.2686	0.2765	0.2850	0.2941
**Scenario 4**					
RD	0.1765	0.1924	0.2016	0.2247	0.2373
RD-RTB	0.1478	0.1614	0.1791	0.2004	0.2164
RTB	0.2378	0.2427	0.2478	0.2538	0.2586

As shown in the following charts (Tables 1–3 and Figs 1-3), we analyzed the RD-RTB method. Compared to the RD and RTB methods, the RD-RTB method shows a certain degree of improvement in bias, standard errors of the least squares means, and rubin’s rules combined standard errors under different scenarios and efficacy levels.

From the above charts (Tables 1–3 and Figs 1-3), we can see that our improved method outperforms the other two methods in terms of performance in all scenarios. As shown in Fig 1, the bias of the RD - RTB method is lower than that of the RTB method and RD method in all scenarios, indicating its more accurate treatment effect estimation. From Fig 2, the RD - RTB method generally has a smaller least squares means standard error, suggesting more precise estimates. Fig 3 shows that the RD - RTB method also has a lower rubin’s rule standard error compared to the RTB method and RD method, validating its effectiveness in reducing errors and providing more reliable results. The RD method is less affected by drug efficacy bias compared to the true efficacy, while the RTB method shows more pronounced fluctuations in bias as the drug efficacy of RD subjects changes, leading to greater bias and a significant underestimation of drug efficacy. Our improved method leverages the correction term from the RTB method but does not exhibit the same large fluctuations with changing drug efficacy. Instead, it shows less bias compared to both methods. The RD method, which does not account for the difference in intercurrent event types between RD subjects and those with missing data, assumes that the outcomes for non-RD subjects are consistent with those of RD subjects. Therefore, its performance in terms of bias is poorer compared to the RD-RTB method. Among the two standard error statistics, the RTB method consistently performs poorly. This might be due to the RTB method’s characteristic of regressing the final imputation results back to the baseline, which results in poor fit for the least squares model, overestimating the least squares mean standard error and increasing the final standard error in rubin’s rule integration. Our improved method enhances the correlation with the baseline in the least squares model fitting, thereby improving the least squares mean standard error and achieving a degree of improvement in rubin’s rule integration standard error compared to the other two methods. The box plots show that the bias of all methods has high dispersion. This is because we simulated 100 datasets, each with different RD subjects, leading to varying bias in the analysis results across different simulated datasets. For the standard error, the analysis results between different datasets do not show high dispersion since the standard error results do not vary significantly with changes in RD subjects.

## 5. Discussion

In clinical trials, it is inevitable that subjects will experience intercurrent events, which may lead to treatment withdrawal due to adverse events, low drug efficacy, or the need for emergency treatment. However, the occurrence of intercurrent events does not necessarily mean that data from subjects who withdraw from treatment will be missing. Researchers often educate these withdrawing subjects about the scientific importance of data collection and make extra efforts to prevent data loss, referring to these subjects who continue to provide data as RD subjects. Currently, assuming missing data is of the MNAR type is becoming a future trend [24], as subjects experiencing intercurrent events often do not maintain the same level of treatment effect after withdrawal treatment. In this case, MNAR-type missing data assumptions become more reliable. The RD method is an imputation method based on MNAR-type assumptions, using RD subjects who have also experienced intercurrent events to impute non-RD subjects, under the assumption that the efficacy of the non-RD subjects is similar to that of the RD subjects. Another MNAR-type missing data handling method is the RTB method, which assumes that after withdrawal treatment, the treatment outcomes will return to baseline levels, effectively losing efficacy. This scenario is often applicable to drugs with very short half-lives or in fields like pain relief, diabetes, or insomnia, where the RTB method’s assumptions are reasonable [25].

However, in reality, there are situations where the efficacy of the non-RD subjects and the RD subjects may not be the same because the type of intercurrent event differs or the efficacy is not returned to baseline after withdrawal treatment. In these cases, since the applicable scenarios are different, both the RD method and the RTB method can lead to bias that deviates from the true efficacy.

Based on these considerations, we have developed a method that can be applied to different scenarios. We have made improvements to the RTB algorithm by weakening the processing of the correction term $\overset{―}{Y_{0}}-\overset{―}{Y_{iJ}}$ in the RTB method, reducing its degree of correction. We also use the parameter ρ to judge whether the efficacy of the missing data subject is superior to that of the RD subject based on the impact of the intercurrent event on the efficacy. The severity of the intercurrent event is judged by the overallefficacy rate before withdrawal treatment, with a lower efficacy rate indicating higher severity of the intercurrent event and the value of ρ being positive. The sign of the correction term sign(ρ) is used to determine whether the correction term is positive or negative, allowing it to flexibly adapt to different scenarios and reducing bias compared to both the RD and RTB methods. Compared to the RTB method, our algorithm significantly reduces the fluctuation of bias caused by changes in drug efficacy and approaches the RD method more closely. In terms of the standard error, our algorithm strengthens the correlation between the imputed efficacy outcomes and the subject’s baseline and the time of treatment withdrawal, improving the fit of the least squares model and reducing both the standard error of the least squares mean and the standard error integrated by rubin’s rule. To verify the applicability and rationality of the improved algorithm, we generated multiple datasets through simulation and analyzed the bias of each dataset. The improved algorithm still exhibits robustness and does not produce obvious outliers. Compared with other methods applied in treatment policy strategies, such as the method by Cro et al. [26], our approach considers the differences in intercurrent events and efficacy between subjects with missing data and RD subjects. Our method can flexibly adapt to different scenarios and bring the efficacy estimates closer to the true values, however, the method by Cro et al. is based on the RBI (reference-based imputation) method as the fundamental imputation model, which can be applied to impute various types of missing data, including Binary, Count, and Ordinal data, our method can not be applied this situation.

In real clinical trials, before selecting an appropriate method for handling missing data as the primary analysis method, it is essential to first consider the medical validity of the method and its applicability within the estimand framework [27]. For example, the RTB method assumes that subjects return to baseline levels after withdrawal treatment, which is applicable to clinical trials involving drugs with short half-lives or pain relief medications. Similarly, the RD method uses complete data from RD subjects to impute non-RD subjects who experience intercurrent events of similar severity, assuming their efficacy after treatment withdrawal is similar. In such cases, using the RD method is often more reasonable. Therefore, when selecting an analysis method, it is crucial to make a reasonable choice based on the specific circumstances of the clinical trial.

Our study focuses on the clinical trial of the effects of antidepressants on the HAMD-17 depression scale scores. However, this method is also applicable to other relevant research fields. It is worth noting that this study is based on the treatment policy strategy. There are certain limitations in the application of other intercurrent event handling strategies. When it comes to other strategies, researchers can consider other methods for handling missing data, such as MMRM (mixed-effects model for repeated measures) and multiple imputation. And when there is limited information collected from subjects after treatment discontinuation, it may introduce bias into the efficacy estimates. Therefore, we recommend that when clinical studies face limited data collection after treatment discontinuation, the RBI method should be used as the core model for imputation to provide robust sensitivity analyses for the study [28]. In addition,although this study only addresses continuous missing data, in the methods for imputing missing data, there are also techniques that can handle discrete data, such as fully conditional specification (FCS), future research could expand the applicability of this method, develop new imputation models, and improve algorithms to handle binary or other types of data. In this study, the improved method we proposed shows some enhancements compared to the RD (retrieved dropout) method and the RTB (return to baseline) method. However, there is still a gap between the results of our method and the true efficacy of the drug.In future, we can continue to develop algorithms to improve the imputation model, so that it can still perform well when dealing with a limited number of subjects who withdraw from treatment and intercurrent events, with smaller bias and standard errors. Since some methods for handling MNAR-type missing data were not included in this study, in future, we plan to further improve our method and we plan to incorporate additional imputation methods for comparison, such as delta-based methods and pattern-mixture models. Additionally, the simulated datasets used in this study only consider the scenario where subjects do not return to the trial after treatment withdrawal. For the scenario where subjects have missing data after treatment withdrawal but may potentially return to the trial and continue treatment and have their data collected, We did not simulate this particular scenario.Future research could provide further validation by addressing these scenarios.

## 6. Conclusion

In this study, we employed a combination of the RD method and the RTB method, incorporating improvements to the RTB formula, to generate a final efficacy analysis that adapts flexibly based on the occurrence of intercurrent events, the result is closer to the true drug efficacy. Our approach resolves the issue of overly conservative imputation results in the RTB method and the absence of consideration for intercurrent events in the RD method. The final analysis results demonstrate that the least squares mean standard error of RD-RTB, the integrated standard error using rubin’s rule, and the bias compared to the true efficacy are all superior to the other two methods. Therefore, based on changes in intercurrent events, in real clinical trial data analysis scenarios,we should select the most scientifically and reasonably appropriate imputation method based on different specific circumstances.

## Supporting information

S1 Data Data provide. (XLSX)
